# Supplementary material for: Analysis of vehicle carbon emission characteristics on expressways in mountainous plateau areas based on the coupled simulation of CarSim/TruckSim and MOVES
Source: PLoS One. 2025 Feb 5;20(2):e0318694. doi: 10.1371/journal.pone.0318694 (PMC11798434; doi:10.1371/journal.pone.0318694)
Supplement: S1 Table — (DOCX) [file pone.0318694.s001.docx]

**S 1 Table.**

| **(a) LDV-CO_2e_ (g/km)** | | | | | | | | |
| --- | --- | --- | --- | --- | --- | --- | --- | --- |
| **S (Acceleration section a)** | **S (Stable driving section)** | **S (Acceleration section b)** | **SS** | **HC (Exit-to-curve)** | **HC (Exit-to-straight)** | **HC (Curved entrance)** | **HC (Straight entrance)** | **CS,LS** |
| 174.36844 | 137.66788 | 148.06646 | 122.6006 | 206.03668 | 182.49123 | 146.9907 | 78.79388 | 167.2971 |
| 138.99782 | 151.30738 | 213.37718 | 146.0452 | 209.3582 | 158.29553 | 129.81549 | 129.45166 | 171.3946 |
| 215.81006 | 147.99421 | 176.22849 | 116.0634 | 159.84712 | 118.76417 | 130.54627 | 74.10982 | 166.3561 |
| 184.30427 | 87.07798 | 200.00235 | 201.7466 | 139.03353 | 171.48829 | 104.05006 | 123.00191 | 163.4919 |
| 189.97585 | 147.71014 | 221.16611 | 176.7093 | 161.11531 | 123.87317 | 136.27469 | 115.61497 | 164.3239 |
| 172.36128 | 155.94549 | 164.84276 | 151.2633 | 164.49779 | 134.37802 | 126.07538 | 111.98723 | 161.8295 |
| 157.10866 | 137.02538 |  |  | 164.19928 | 170.2022 | 113.04746 | 108.84662 | 173.3891 |
| 159.71494 | 115.29709 |  |  | 160.62151 | 163.76878 | 85.16882 | 125.74427 | 165.8953 |
| 174.01964 | 127.85379 |  |  | 161.74213 | 123.76166 | 119.36415 | 112.51096 | 196.5453 |
| 145.64963 | 155.98452 |  |  | 162.39779 | 151.41981 | 119.76288 | 122.73316 | 146.2079 |
| 148.74935 | 167.60249 |  |  | 169.82158 | 198.39893 | 123.97318 | 112.01641 | 182.2751 |
| 159.75623 | 140.54776 |  |  | 120.9865 | 155.29744 | 72.58084 | 123.70742 | 171.5231 |
| 178.49917 | 155.31121 |  |  | 145.62329 | 121.96098 | 137.99422 | 131.18092 | 203.9412 |
| 221.52269 | 125.79232 |  |  | 124.07922 | 146.95464 | 79.86007 | 131.90934 | 167.7533 |
| 138.55484 | 154.98713 |  |  | 177.65267 | 132.76285 | 112.50963 | 96.44091 | 182.2432 |
| 134.64482 | 137.19173 |  |  | 139.73063 | 153.83612 | 91.33397 | 135.21173 |  |
| 201.6157 | 137.83728 |  |  | 225.87641 | 156.60286 | 146.76357 | 105.17263 |  |
| 138.16981 | 149.19373 |  |  | 229.64 | 170.7318 | 159.28114 | 119.17379 |  |
| 168.62633 | 144.80084 |  |  | 176.87605 | 124.44332 | 131.0048 | 124.44355 |  |
| 134.64482 | 156.10868 |  |  | 165.6627 | 163.60126 | 163.18044 | 118.63775 |  |
| 138.80178 | 136.55361 |  |  | 181.37593 | 125.28713 | 152.29325 | 78.1802 |  |
| 207.47016 | 154.53689 |  |  | 220.33174 | 151.65136 | 120.59318 | 123.32827 |  |
| 148.80231 | 156.87019 |  |  | 183.01077 | 163.72777 | 119.07116 | 109.31335 |  |
| 214.27449 | 151.94688 |  |  | 212.34101 | 163.48185 | 163.31121 | 129.30491 |  |
| 208.19516 | 167.89562 |  |  |  | 147.57599 |  | 129.49907 |  |
| 144.28459 | 150.12812 |  |  |  | 183.42602 |  | 119.49106 |  |
| 190.48133 | 152.31639 |  |  |  | 155.97795 |  | 148.45542 |  |
| 198.43979 | 161.20726 |  |  |  | 144.42176 |  | 112.45082 |  |
| 181.62842 | 154.019 |  |  |  | 172.41904 |  | 126.96392 |  |
| 188.601 | 199.60501 |  |  |  | 167.35224 |  | 128.49628 | -- |
| 209.0901 | 187.94425 |  |  |  | 154.04763 |  | 83.88507 |  |
| 216.70146 | 137.64395 |  |  |  | 161.1503 |  | 71.08926 |  |
| 200.89 | 158.10026 |  |  |  | 144.66783 |  | 111.07878 |  |
| 209.24258 | 179.43786 |  |  |  | 155.20041 |  | 115.57147 |  |
| 203.40204 | 156.96526 |  |  |  | 167.46445 |  | 116.91867 |  |
| 207.47016 | 158.88517 |  |  |  | 207.68754 |  | 128.7462 |  |
| 175.56373 |  |  |  |  | 207.69785 |  | 166.35669 |  |
|  |  |  |  |  | 162.28649 |  | 135.80883 |  |
|  |  |  |  |  | 141.68944 |  | 118.06183 |  |
|  |  |  |  |  | 163.18044 |  | 73.02071 |  |
|  |  |  |  |  | 166.51373 |  | 127.19901 |  |
|  |  |  |  |  | 212.31246 |  | 156.23514 |  |
|  |  |  |  |  | 196.352 |  | 116.65515 |  |
|  |  |  |  |  | 215.91082 |  | 134.89502 |  |
|  |  |  |  |  | 172.02877 |  | 124.79022 |  |
|  |  |  |  |  | 182.24234 |  | 120.07909 |  |
|  |  |  |  |  | 204.67136 |  | 75.15826 |  |

| **(b) LDV-CO_2e_ (g/km)** | | | | | | | | |
| --- | --- | --- | --- | --- | --- | --- | --- | --- |
| **S (Acceleration section a)** | **S (Stable driving section)** | **S (Acceleration section b)** | **SS** | **HC (Exit-to-curve)** | **HC (Exit-to-straight)** | **HC (Curved entrance)** | **HC (Straight entrance)** | **CS、LS** |
| 703.4496 | 194.88808 | 918.30056 | 433.1736 | 592.92541 | 793.81315 | 853.5899 | 436.39058 | 666.38083 |
| 1202.05831 | 713.93066 | 880.28996 | 858.5802 | 845.64596 | 605.43655 | 772.98275 | 739.21457 | 1168.54297 |
| 1027.5753 | 615.44143 | 1430.68571 | 686.2984 | 598.73583 | 400.66753 | 618.70223 | 400.66753 | 760.89826 |
| 792.39623 | 716.00298 | 1255.68306 | 878.672 | 320.53834 | 902.46679 | 483.22099 | 592.92843 | 1200.28928 |
| 1041.73368 | 703.92842 | 1059.06641 | 841.8336 | 579.6667 | 507.84569 | 664.79342 | 585.98264 | 913.27862 |
| 719.62721 | 442.43676 |  | 809.7247 | 568.92138 | 534.83152 | 639.00016 | 590.50536 | 1024.42525 |
| 1083.78339 | 622.52678 |  |  | 702.90019 | 743.39228 | 583.12093 | 521.95512 | 970.25251 |
| 827.96796 | 740.29597 |  |  | 444.75528 | 632.876 | 649.18536 | 613.42248 | 812.73068 |
| 701.14225 | 665.00187 |  |  | 627.09453 | 449.91574 | 625.11167 | 598.76286 | 1006.84472 |
| 1006.18013 | 647.63463 |  |  | 478.19621 | 594.05535 | 598.06273 | 598.73331 | 918.07768 |
| 763.00432 | 731.74197 |  |  | 647.6624 | 842.34702 | 639.76994 | 624.51811 | 998.82332 |
| 673.32072 | 446.22819 |  |  | 167.31701 | 746.30889 | 368.54178 | 773.16283 | 1121.33467 |
| 972.72662 | 684.96796 |  |  | 430.68698 | 480.77351 | 699.40957 | 629.47944 | 1036.9383 |
| 1121.80754 | 583.42915 |  |  | 273.78859 | 626.0357 | 481.20074 | 773.80583 | 924.5621 |
| 1267.86204 | 751.54242 |  |  | 463.57705 | 531.74997 | 627.26792 | 468.95066 | 1004.42234 |
| 912.93813 | 587.68726 |  |  | 711.71334 | 702.76899 | 684.06678 | 753.65474 |  |
| 742.73263 | 506.42584 |  |  | 661.51131 | 849.32588 | 859.09496 | 568.80868 |  |
| 1252.11231 | 623.85479 |  |  | 673.77902 | 622.90884 | 864.82211 | 591.93978 |  |
| 869.90468 | 671.43471 |  |  | 722.74998 | 525.35991 | 724.2032 | 588.68086 |  |
| 912.78368 | 585.21507 |  |  | 631.80134 | 801.49192 | 850.859 | 549.11861 |  |
| 1021.53264 | 589.55853 |  |  | 792.19921 | 414.54524 | 879.32428 | 438.93025 |  |
| 1115.93072 | 695.58574 |  |  | 713.94889 | 652.84214 | 915.65573 | 570.2602 |  |
| 867.65273 | 793.57699 |  |  | 640.98651 | 647.69503 | 683.88807 | 628.1786 |  |
| 1129.03124 | 930.57131 |  |  | 754.29233 | 837.88709 | 741.46826 | 675.62656 |  |
| 1102.03202 | 736.7153 |  |  |  | 787.14786 |  | 574.30877 |  |
| 1327.1399 | 848.88216 |  |  |  | 871.26364 |  | 581.68276 |  |
| 1124.46534 | 699.20173 |  |  |  | 688.8786 |  | 761.64588 |  |
| 1087.25924 | 687.38899 |  |  |  | 693.7045 |  | 555.14117 |  |
| 912.76639 | 791.75238 |  |  |  | 783.5831 |  | 745.86389 |  |
| 1045.2467 | 654.30725 |  |  |  | 903.02247 |  | 689.55028 |  |
| 959.747 | 793.34496 |  |  |  | 723.80463 |  | 239.01921 |  |
| 1439.1823 | 995.7553 |  |  |  | 696.1599 |  | 360.59539 |  |
| 1441.67914 | 1137.78033 |  |  |  | 688.28132 |  | 551.7316 |  |
| 1304.38408 | 880.91622 |  |  |  | 532.04964 |  | 651.65396 |  |
| 1173.15531 | 659.21941 |  |  |  | 726.05983 |  | 533.25409 |  |
| 1301.65513 | 802.68102 |  |  |  | 973.72816 |  | 639.1195 |  |
| 1287.84633 | 916.94877 |  |  |  | 940.35822 |  | 812.00262 |  |
| 1115.52286 | 795.25388 |  |  |  | 936.95523 |  | 642.44442 |  |
| 1292.29939 | 777.57355 |  |  |  | 659.25677 |  | 683.09093 |  |
|  |  |  |  |  | 935.53846 |  | 432.73887 |  |
|  |  |  |  |  | 836.03739 |  | 663.28265 |  |
|  |  |  |  |  | 941.70589 |  | 854.25861 |  |
|  |  |  |  |  | 895.73018 |  | 750.73467 |  |
|  |  |  |  |  | 902.03178 |  | 829.95614 |  |
|  |  |  |  |  | 804.47066 |  | 763.39311 |  |
|  |  |  |  |  | 831.25753 |  | 715.31316 |  |
|  |  |  |  |  | 848.36072 |  | 620.5546 |  |
